# RasGRP3 regulates the migration of glioma cells via interaction with Arp3

**DOI:** 10.18632/oncotarget.2575

**Published:** 2015-02-11

**Authors:** Hae Kyung Lee, Susan Finniss, Simona Cazacu, Cunli Xiang, Laila M. Poisson, Peter M. Blumberg, Chaya Brodie

**Affiliations:** ^1^ Davidson Laboratory of Cell Signaling and Tumorigenesis, Department of Neurosurgery, Hermelin Brain Tumor Center, Henry Ford Hospital, Detroit, MI, USA; ^2^ Hermelin Brain Tumor Center, Henry Ford Hospital, Detroit, MI, USA; ^3^ Department of Public Health Sciences, Center for Bioinformatics, Henry Ford Hospital, Detroit, MI, USA; ^4^ Laboratory of Cancer Biology and Genetics, Center for Cancer Research, National Cancer Institute, Bethesda, MD, USA; ^5^ Everard and Mina Goodman Faculty of Life Sciences, Bar-Ilan University, Ramat-Gan, Israel

## Abstract

Glioblastoma (GBM), the most aggressive primary brain tumors, are highly infiltrative. Although GBM express high Ras activity and Ras proteins have been implicated in gliomagenesis, Ras-activating mutations are not frequent in these tumors. RasGRP3, an important signaling protein responsive to diacylglycerol (DAG), increases Ras activation. Here, we examined the expression and functions of RasGRP3 in GBM and glioma cells. RasGRP3 expression was upregulated in GBM specimens and glioma stem cells compared with normal brains and neural stem cells, respectively. RasGRP3 activated Ras and Rap1 in glioma cells and increased cell migration and invasion partially via Ras activation. Using pull-down assay and mass spectroscopy we identified the actin-related protein, Arp3, as a novel interacting protein of RasGRP3. The interaction of RasGRP3 and Arp3 was validated by immunofluorescence staining and co-immunoprecipitation, and PMA, which activates RasGRP3 and induces its translocation to the peri-nuclear region, increased the association of Arp3 and RasGRP3. Arp3 was upregulated in GBM, regulated cell spreading and migration and its silencing partially decreased these effects of RasGRP3 in glioma cells. In summary, RasGRP3 acts as an important integrating signaling protein of the DAG and Ras signaling pathways and actin polymerization and represents an important therapeutic target in GBM.

## INTRODUCTION

Glioblastoma (GBM), the most malignant of the primary brain tumors, are characterized by increased proliferation and invasion into the surrounding normal brain tissue [1]. Limitations to therapy are mainly due to the infiltrative nature of the tumors which prevents complete resection and contributes to tumor recurrence and the high resistance to radio- and chemotherapy of residual tumor cells and glioma stem cells (GSCs) [2, 3]. Understanding the mechanisms that regulate glioma cell migration is thus crucial for the development of novel effective interventions.

Recently, gene expression profiling has identified five GBM subtypes, which are classified based on their transcriptional signatures into proneural, G-CIMP, neural, classical and mesenchymal subtypes [4, 5]. These subtypes have distinct differential genetic alterations, molecular signature, and cellular phenotypes and are associated with different degree of infiltration and poor patient survival. In particular, the mesenchymal subtype of GBM is characterized by an increased level of infiltration, resistance to radiation and poor prognosis. Moreover, recurrent tumors tend to express mesenchymal phenotypes.

The RasGRP family of guanine nucleotide exchange factors (GEFs) activate small GTPases including Ras and Rap1 [6]. RasGRP activation is controlled both by membrane recruitment through a DAG binding C1 domain and by PKC-dependent phosphorylation [7–9]. Signaling pathways coupled to DAG generation are highly active in glioma, mainly downstream of activated epidermal growth factor (EGF) and platelet-derived growth factor (PDGF) receptors [10, 11]. RasGRP3 is one of four members of the RasGRP family [12, 13]. While the different RasGRP proteins generally share similar mechanisms of regulation, they exhibit distinct patterns of tissue expression and specificity for Ras and Rap GTPases [12, 14–16]. The role of the RasGRP proteins in carcinogenesis and malignant transformation is just beginning to be understood. Recent studies have reported that RasGRPs can function as oncogenes in multiple cancers, inducing tumorigenesis in both mouse models and in humans [17–19], Elevated RasGRP3 expression is found in human prostate cancer and human melanoma and has been implicated in their tumorigenicity [20, 21]. The ability of the RasGRP proteins to bind DAG and to modulate Ras activity allows them to directly link the DAG/phorbol ester signaling with the Ras pathway and the malignant transformation process.

GBM express hyperactive Ras and Rap1, but Ras and Rap1 mutations are rare in these tumors [22, 23]. In the present study we characterized the expression and functions of RasGRP3 in GBM specimens and glioma cells, examined the role of RasGRP3 in the activation of Ras and Rap1, and studied the signaling pathways that mediate its effects. We found that RasGRP3 is highly expressed in mesenchymal GBM and is involved in the cell migration and invasion of glioma cells and the regulation of Ras activity. In addition, we identified actin-related protein 3 (Arp3), as a novel interacting protein of RasGRP3 and characterized its contribution to RasGRP3 functions.

## RESULTS

### RasGRP3 expression in GBM, glioma cells and GSCs

**Figure 1:**
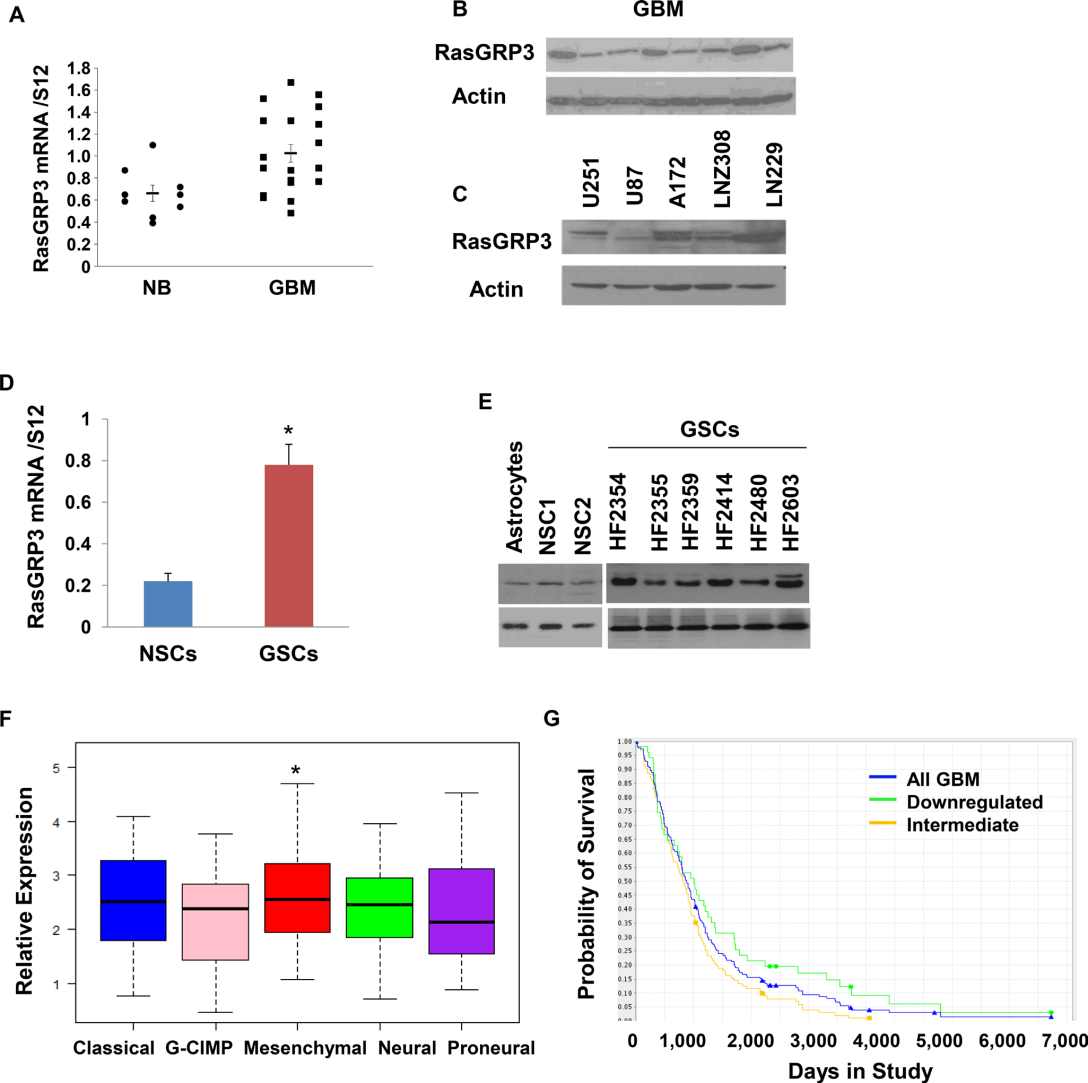
Expression of RasGRP3 in GBM, glioma cell lines and GSCs Total RNA was extracted from normal brains (NB) and GBM specimens and the expression of RasGRP3 was determined using real-time PCR **(A)**. Data from individual human tissues are presented with the median and interquartile range noted. Age adjusted *t*-test, *P* = 0.001. Results are normalized relative to the levels of S12 mRNA and are presented relative to a reference sample (*p* < 0.01 for all tumors as compared to non-tumor specimens). Protein extracts from GBM specimens **(B)** or glioma cell lines **(C)** were examined for the expression of the RasGRP3 protein using Western blot analysis. The expression of RasGRP3 in twelve different primary GSCs that were generated from GBM specimens and in human NSCs was measured using real-time PCR **(D)** or by Western blot analysis **(E)**. Gene expression plot presenting five GBM subtypes of 496 primary GBM patients from TCGA showing differential expression of RasGRP3 gene (probe A_23_P209544, ANOVA F-test, *P* < 0.005). Mesenchymal samples are significantly higher on average than the GCIMP and proneural groups (*t*-test, *P* < 0.05) **(F)**. Kaplan-Meier plot of overall survival stratified by RasGRP3 gene expression level for 165 GBM patients from REMBRANDT with differential RasGRP3 gene expression (log-rank *P* < 0.005) **(G)**. Increased levels of RasGRP3 are correlated with poor prognosis.

We first examined the expression of RasGRP3 in GBM using RT-PCR and Western blot analysis. We found that GBM tumors expressed RasGRP3 mRNA (Fig. 1A) and protein (Fig. 1B) and that the expression of RasGRP3 mRNA was higher in GBM compared to normal brain (*P* < 0.009). The expression of RasGRP3 was also examined in glioma cell lines. Among the cell lines that were examined, A172, U251 and LNZ308 expressed the highest levels of RasGRP3, whereas the U87 cells expressed the lowest level (Fig. 1C).

GBM contain a small population of GSCs, which have been implicated in the resistance to therapy and recurrence of these tumors [24]. Using 12 lines of GSCs that were generated from fresh GBM specimens as described previously [25], we found that the expression of RasGRP3 in these cells was significantly higher than that of human neural stem cells (NSCs) (Figs. 1D and 1E).

Using the TCGA data portal [26], we analyzed the relative expression of RasGRP3 in the five GBM subtypes. We found that the mean expression of RasGRP3 was significantly higher in tumors that are classified as the mesenchymal subtype (*p* < 0.05) compared to the proneural, neural or G-CIMP GBM subtypes (Fig. 1F).

We further examined the expression of RasGRP3 in GBM by using the REMBRANDT (Repository of Molecular Brain Neoplasia Data) data portal and found that intermediate levels of RasGRP3 were significantly (*p* < 0.005) associated with a worse clinical outcome compared to tumors with downregulated levels of this protein (Fig. 1G).

### Role of RasGRP3 in the migration and invasion of glioma cells

**Figure 2:**
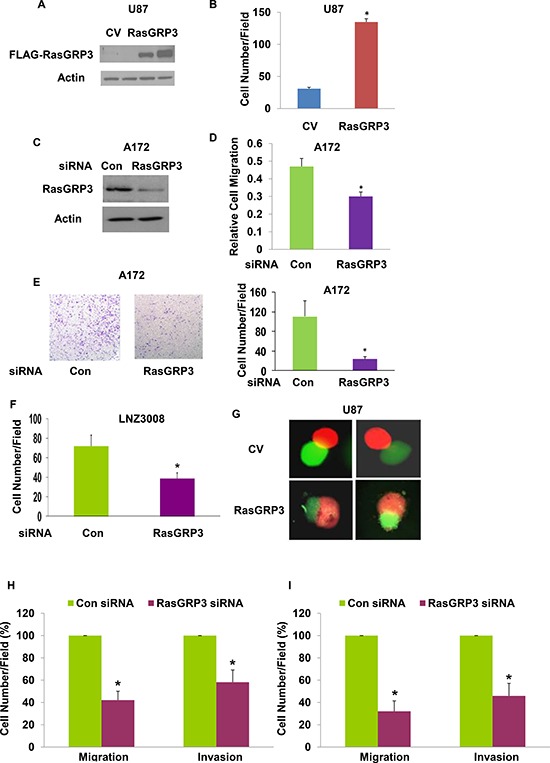
RasGRP3 regulates the migration and invasion of glioma cells Expression of exogenous RasGRP3 in stable clones of U87 is shown in a Western blot compared with control vector cells (CV) using anti-FLAG antibody **(A)**. Cell migration was assayed using a transwell chamber containing polycarbonate membrane inserts of 8-μm pore size (Corning, Costar) with fibronectin coating. U87 cells overexpressing RasGRP3 (3 × 10^4^ cells) in serum-free DMEM were added to the upper chamber. The bottom chamber was filled with 600 μl of serum-containing medium, and the assembly was incubated at 37ºC for 3–4 hrs to allow cell migration. The cells were removed from the top of the membrane and the migratory cells were stained and quantified. Values shown are the mean ± SE of triplicate experiments **(B)**. A172 cells were transfected with control or RasGRP3 siRNA pools and the expression of the RasGRP3 protein was determined by Western blot analysis **(C)**. Cell migration was determined as described in Methods **(D)**. The results represent the means ± SE of three independent experiments (*P* < 0.01). A172 cells transfected with control siRNA or RasGRP3 siRNAs were plated onto BD invasion chambers (8-μm pore size with polycarbonate membranes). Matrigel invasion assays of A172 cells transfected with control or RasGRP3 siRNAs were performed as described in Methods **(E)**. Representative images are shown. The results were quantitated and the graph presents the means ± SE of three independent experiments (*P* < 0.01). Matrigel invasion assay was determined also for the LNZ308 glioma cells transfected with either control or RasGRP3 siRNAs **(F)**. In the confrontation assay, spheroids of U87 cells overexpressing RasGRP3 or a control vector labeled with Green Cell Tracker were confronted with astrocytic spheroids labeled with Red Cell Tracker and cell invasion was determined after 3 days **(G)**. **(H–I)** The effect of RasGRP3 silencing was also examined on the migration (transwell assay) and invasion (matrigel invasion assay) in the HF2354 (H) and HF2607 (I) GSCs. For these experiments, GSC neurospheres that were silenced for RasGRP3 for 3 days were disaggregated and analyzed for the migration and invasion assays as described for the glioma cell lines. The results represent three different experiments, each consisted of 6 cultures that gave similar results. *p < 0.005

To study the role of RasGRP3 in glioma cell migration, we employed two approaches. In the first we stably overexpressed RasGRP3 in the U87 cells that expressed low levels of the endogenous protein and examined the migration of these cells as compared to the CV cells. Western blot analysis confirmed overexpression of RasGRP3 in two stable clones of U87 cells (Fig. 2A). The effect of RasGRP3 on glioma cell migration was analyzed using the transwell migration assay and the results of this assay indicated that RasGRP3 overexpression significantly increased the migration of these cells compared to the CV cells (Fig. 2B) (*p* < 0.01).

We further examined the role of the endogenous RasGRP3 protein in glioma cell migration by silencing RasGRP3 in the A172 cells which express high levels of this protein. Transfection of A172 with a siRNA duplex for 3 days resulted in about 80% decrease in the expression of the RasGRP3 protein, compared to cells transfected with control siRNA (Fig. 2C). The RasGRP3-silenced A172 cells exhibited a lower degree of cell migration compared with the control siRNA transfected cells (Fig. 2D) (*p* < 0.01), further indicating that RasGRP3 plays a role in glioma cell migration.

Using the Boyden chamber matrigel invasion assay, we also evaluated the invasion ability of A172 cells silenced for RasGRP3 and found that these cells exhibited about a 5-fold decrease in cell invasion as compared to control A172 cells (Fig. 2E) (*p* < 0.01). Similar results were obtained for the LNZ308 glioma cells (Fig. 2F) and for two GSCs (Figs. 2H and 2I).

Finally, we examined the role of RasGRP3 on glioma cell invasion using the spheroid confrontation assay as previously described [27]. In this assay, spheroids of U87 cells overexpressing RasGRP3 or a control vector labeled with Green Cell Tracker are confronted with astrocytic spheroids labeled with Red Cell Tracker Green. Following confrontation, the spheroids are observed in a fluorescence microscope and the degree of cell invasion is evaluated. The U87 spheroids that overexpressed RasGRP3 invaded to a larger degree the astrocytic spheroids as compared to the U87 spheroids that expressed a CV (Fig. 2G). Similar results were obtained in additional three experiments (data not shown). Thus, overexpression of RasGRP3 increases the invasive responses of glioma cells.

In addition to its effects on glioma cells, we also found that silencing of RasGRP3 decreased the migration and invasion of the HF2354 (Fig. 2H) and HF2607 GSCs (Fig. 2I) that express high levels of this protein.

### RasGRP3 regulates both Ras and Rap1 activities

**Figure 3:**
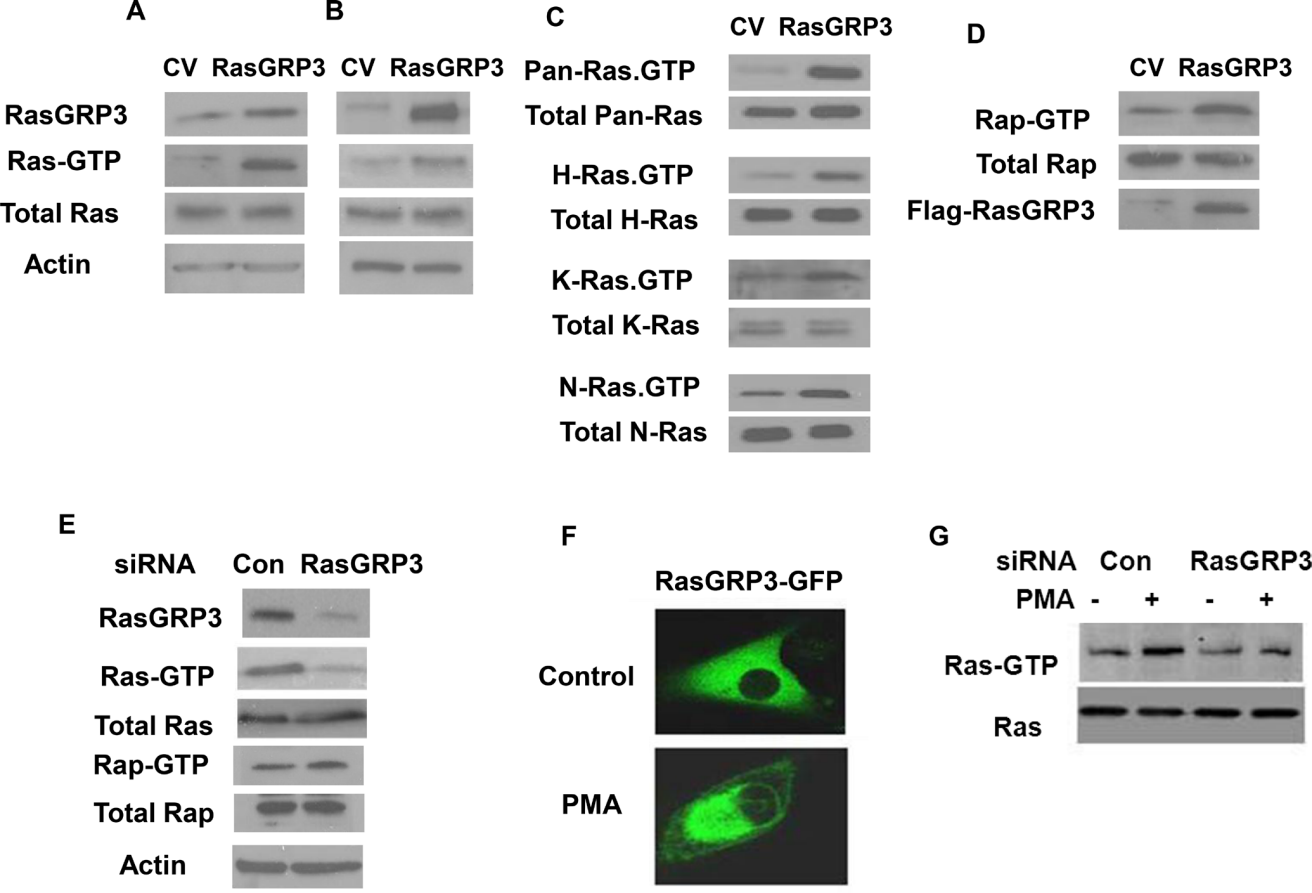
Effect of RasGRP3 on Ras and Rap1 activation Overexpression of RasGRP3 in the U87 cells upregulates active Ras in both U87 stable clones **(A)** and in transiently overexpressing cells **(B)**. Ras and Ras-GTP levels in CV or RasGRP3 overexpressing cells were examined. Lysates of CV or RasGRP3 overexpressing cells were subjected to SDS-PAGE followed by immunoblotting with anti-Ras antibody (total Ras) or to the GST-RBD pull-down assay followed by SDS-PAGE and immunoblotting with anti-Ras antibody for determination of Ras-GTP as described in Methods. All three Ras isoforms (H-, K-, and N-Ras) exhibited enhanced activation upon overexpression of RasGRP3 in the U87 cells, while the levels of the total protein for each isoform were not affected **(C)**. The level of active Rap1 increased in RasGRP3 overexpressing U87 cells compared to control cells while the level of total Rap1 was not affected **(D)**. Silencing of RasGRP3 expression decreased activation of Ras but not of Rap1 in the A172 cells **(E)**. After 3 days of transfection with control and RasGRP3 siRNAs, cells were lysed and incubated with GST-Raf1-RBD (for active Ras) or GST-RalGDS-RBD (for active Rap1). Proteins bound to the beads (GTP-Ras) and total lysates (Ras) were analyzed by immunoblotting with anti-Ras antibody or anti-Rap1. Samples of total lysates were also immunoblotted with anti-actin antibody (E). PMA induced translocation of RasGRP3-GFP to the perinuclear region in the A172 cells as determined by confocal microscopy **(F)**. PMA treatment increased Ras activity whereas silencing of RasGRP3 partially decreased the activation of Ras induced by PMA **(G)**. All results are representative of three experiments that gave similar results.

RasGRP3 acts as a GEF (guanine nucleotide exchange factor) for Ras and Rap1. To examine whether RasGRP3 contributes to the Ras pathway in glioma cells, we analyzed the effect of overexpression of RasGRP3 on Ras activation in the U87 cells. Activation of Ras was measured in cell lysates by pull-down of GTP-Ras as described in the methods. As shown in Fig. 3A, overexpression of RasGRP3 in stable clones of RasGRP3 overexpressing U87 cells significantly increased Ras activity (Ras-GTP), whereas the levels of total Ras were not altered. Similar results were obtained in U87 cells transiently transfected with RasGRP3 for 2 days (Fig. 3B). Analysis of the activity of the different Ras isoforms demonstrated that overexpression of RasGRP3 increased the activity of each of the H-Ras, K-Ras, and N-Ras isoforms expressed in the U87 cells (Fig. 3C). Since Rap1 is another downstream target of RasGRP3, we also examined the activation of Rap1 in RasGRP3 overexpressing cells and found that, similar to Ras, the level of active Rap1 was also increased in the RasGRP3 overexpressing cells compared to control cells (Fig. 3D).

In parallel with the analysis of the effect of overexpressing RasGRP3 in the U87 glioma cells, we examined the effect of RasGRP3 silencing in the A172 cells. These cells were transfected with control or RasGRP3-specific siRNAs, and activation of Ras was analyzed in the cell lysates after 3 days of transfection. As shown in Fig. 3E, silencing of RasGRP3 significantly decreased Ras activity (Ras-GTP), whereas the levels of total Ras were not altered. Interestingly, Rap activity was not significantly altered in the RasGRP3 silenced cells (Fig. 3E).

PMA has been reported to induce Ras activation [7]. Since PMA activates both PKC isoforms and the RasGRP3 protein via binding to the C1 domain, we examined the role of RasGRP3 in the activation of Ras by PMA. As presented in Fig. 3F, PMA induced translocation of RasGRP3 to the peri-nuclear region in the A172 cells. In addition, PMA increased Ras activity in these cells and silencing of RasGRP3 partially decreased the activation of Ras induced by PMA (Fig. 3G).

### Roles of Ras and AKT in RasGRP3-induced glioma cell migration

**Figure 4:**
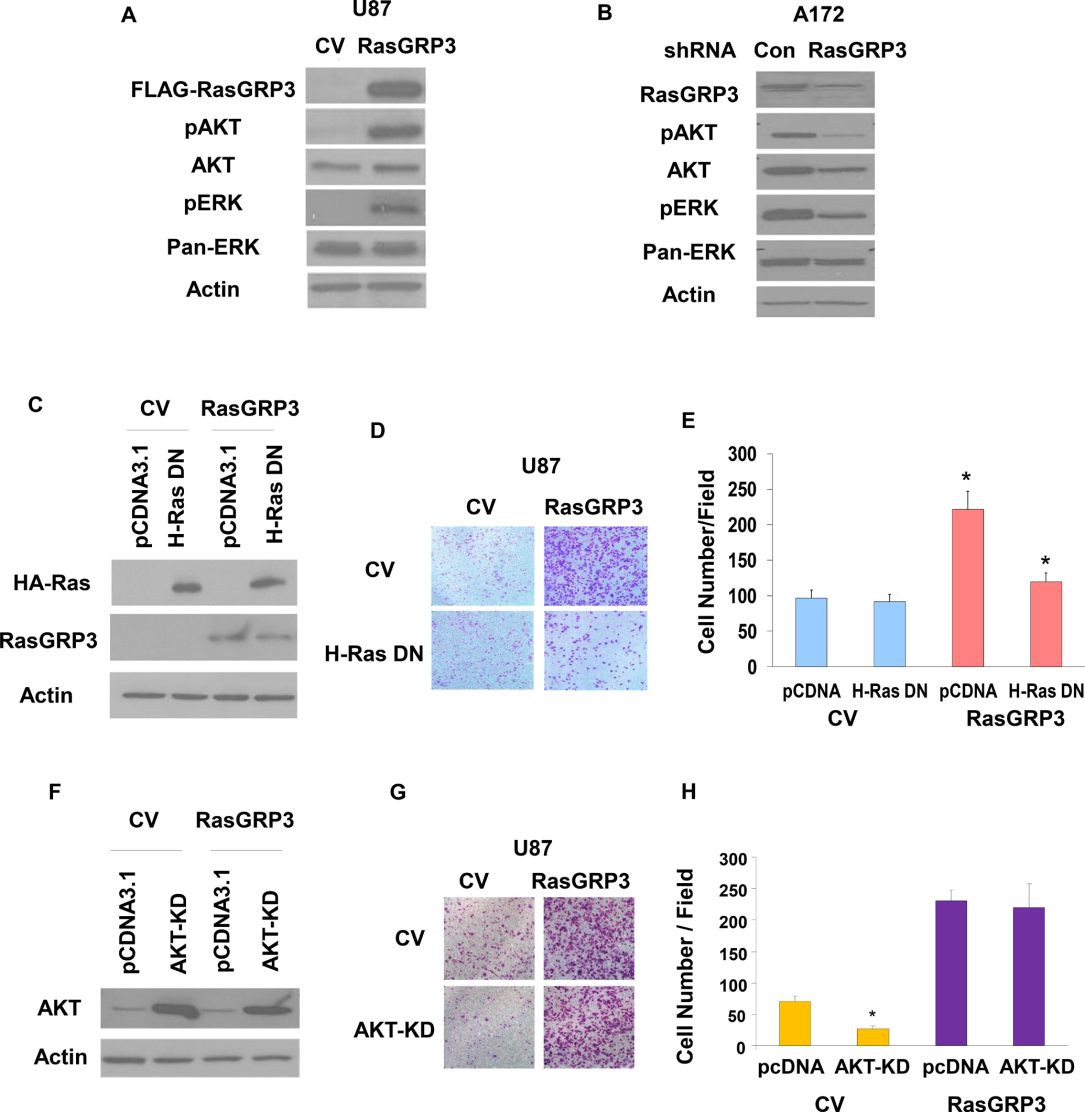
Roles of Ras and AKT activation in RasGRP3-induced glioma cell migration U87 cells overexpressing CV and RasGRP3 **(A)** and A172 cells treated with control shRNA or shRNA targeting RasGRP3 **(B)** were analyzed for the expression and phosphorylation of AKT and ERK1/2 (A,B). Results represent one of three separate experiments that gave similar results. U87 overexpressing CV and RasGRP3 were transfected with H-Ras17N-HA or pcDNA3.1 (control) and the expression of Ras-DN was analyzed using Western blot **(C)**. Transwell migration was performed 72 hrs later, migrating cells were photographed **(D)**, and the number of cells was determined **(E)**. The results represent the means ± S.E of three independent experiments. *P < 0.001. U87 cells overexpressing CV and RasGRP3 were transfected with AKT-KD mutant or pcDNA3.1 (control) and the expression of AKT-KD was analyzed using Western blot **(F)**. Transwell migration was performed 72 hrs later; cells were photographed **(G)**, and their number was determined **(H)**. The results represent the means ± SE of three independent experiments. *P < 0.01

To further study the role of RasGRP3 in glioma cell signaling we examined the effect of RasGRP3 overexpression on two of the Ras downstream signaling pathways, AKT and Erk1/2. RasGRP3 overexpression in the U87 cells led to a substantial increase in the phosphorylation of both AKT and Erk1/2 (Fig. 4A). Conversely, silencing of RasGRP3 in the A172 cells decreased the phosphorylation of AKT and Erk1/2 as compared to the CV cells (Fig. 4B).

To delineate the mechanisms underlying the effects of RasGRP3 on glioma cell migration, we examined the role of the Ras pathway in this process. We overexpressed the Ras DN mutant, RasN17, in the U87 cells transfected with CV or RasGRP3 (Fig. 4C). Transfection of U87 cells overexpressing RasGRP3 with RasN17 significantly decreased the migration of the cells compared to the control cells, whereas no decrease in cell migration was observed in the CV cells (Figs. 4D and 4E). These results strongly implicate the Ras signaling pathway as a mediator of the increased cell migration in the RasGRP3 overexpressing cells.

To examine the role of AKT in the migration of glioma cells we employed an AKT kinase-dead (KD) mutant (Fig. 4F). Overexpression of this mutant decreased the migration of the CV cells, whereas it did not significantly interfere with the increased migration of the RasGRP3 overexpressing cells (Figs. 4G and 4H), suggesting that activation of AKT does not play a role in the increased cell migration induced by RasGRP3.

### Identification of Arp3 as a novel RasGRP3 interacting protein

**Figure 5:**
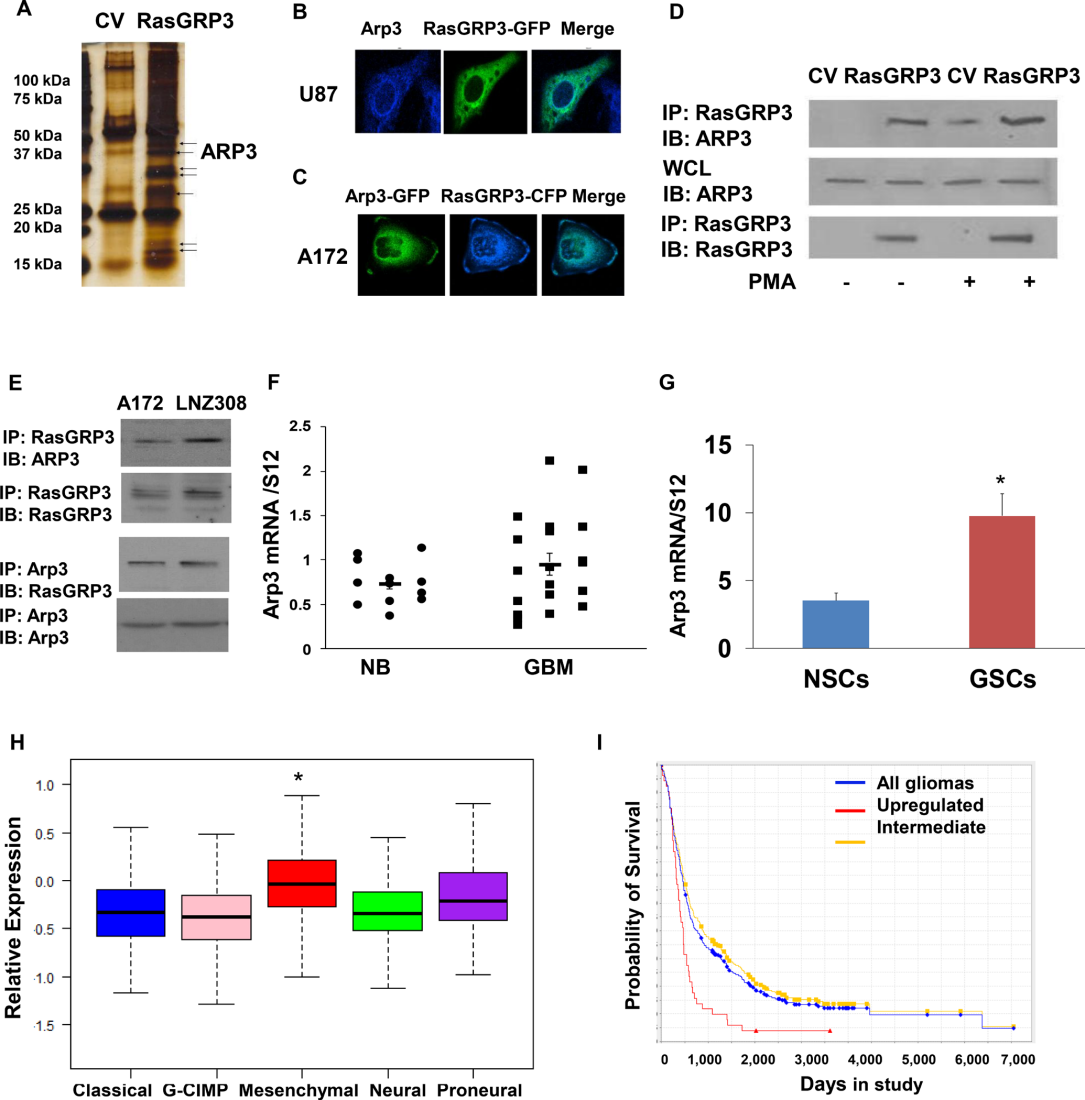
Arp3 is a novel RasGRP3 interacting protein A pull-down assay was performed to identify RasGRP3 partner proteins. The immunoprecipitated complexes were resolved and stained **(A)**. Arp3 was one of the pull-down complexes identified using mass spectrometry analysis. Both RasGRP3 and Arp3 were localized to the cytoplasm as well as to the perinuclear membrane as analyzed by confocal microscopy **(B, C)**. A172 cells were transfected with FLAG-RasGRP3 or with a CV and the cells were incubated with or without PMA (0.1 μM) for 30 min. RasGRP3 was immunoprecipitated using an anti-FLAG antibody and immunoblotted with anti-Arp3 antibody in the A172 cells **(D)**. Immunoprecipitation was also performed for endogenous RasGRP3 and Arp3 **(E)**. Total RNA was extracted from normal brains (NB) and GBM specimens and the expression of Arp3 was determined using real-time PCR **(F)**. Data from individual human tissues are presented with the median and interquartile range noted. Age adjusted *t*-test, *P* = 0.001. Results are normalized relative to the levels of S12 mRNA and are presented relative to a reference sample (*p* < 0.005 for all tumors as compared to non-tumor specimens) (F). The expression of Arp3 in human NSCs and in twelve different primary GSCs that were generated from GBM specimens was measured using real-time PCR **(G)**. Related data were analyzed with *t*-test between two groups. The results represent the means ± SE. *P* < 0.01. Arp3 expression was examined in different subtypes of GBM. The gene expression plot presenting five GBM subtypes of 496 primary GBM specimens from TCGA showing differential Arp3 gene expression **(H)**. (Probe A_23_P108785, ANOVA F-test, *P* < 0.0001). The classical, GCIMP, and neural groups have significantly lower average Arp3 gene expression than the mesenchymal tumors (*t*-test *P* < 0.05). Kaplan-Meier plot of overall survival for glioma patients from REMBRANDT stratified by Arp3 gene expression (log-rank *P* = 0.001). Upregulation of Arp3 expression is correlated with poor patient prognosis **(I)**.

To identify novel binding partners of RasGRP3 in glioma cells, we employed the affinity pull-down assay. Lysates from FLAG-RasGRP3 overexpressing U87 cells or CV cells were immunoprecipitated with anti-FLAG antibody and the immunoprecipitated proteins were analyzed using mass spectrometry. As illustrated in Fig. 5A, some potential RasGRP3-interacting proteins were detected by the pull-down assay. Among the identified proteins, we focus in this report on Arp3, since this protein plays major roles in actin polymerization and cell spreading and migration.

We first examined the localization of RasGRP3 and Arp3 in glioma cells using confocal microscopy. For these experiments, we transfected U87 cells with RasGRP3 fused to GFP and stained them with anti-Arp3 antibody. Both RasGRP3 and Arp3 were localized to the cytoplasm as well as to the peri-nuclear membrane (Fig. 5B). Co-localization was similarly seen in A172 cells transfected with Arp3-GFP and RasGRP3-CFP (Fig. 5C).

To validate the association of RasGRP3 with Arp3, we performed co-immunoprecipitation assays. As presented in Fig. 5D, the two proteins were associated in U87 cells overexpressing RasGRP3 cells and their interaction was further increased following PMA treatment. Similar results were obtained when we performed co-immunoprecipitation of endogenous Arp3 and RasGRP3 in the A172 cells (Fig. 5E).

We then examined the expression of Arp3 in GBM specimens and glioma cell lines and found that the expression of Arp3 was increased in these tumors as compared to normal brain specimens (Fig. 5F) and that it was also expressed in higher levels in GSCs compared to NSCs (Fig. 5G). Similar to the results obtained with RasGRP3, the expression of Arp3 was also increased in the mesenchymal compared to other GBM subtypes (Fig. 5H). Analysis of patient survival based on the REMBRANDT portal revealed that the expression of Arp3 in glioma was inversely correlated with patient survival (Fig. 5I).

### Arp3 plays a role in the effects of RasGRP3 on glioma cell spreading and migration

**Figure 6:**
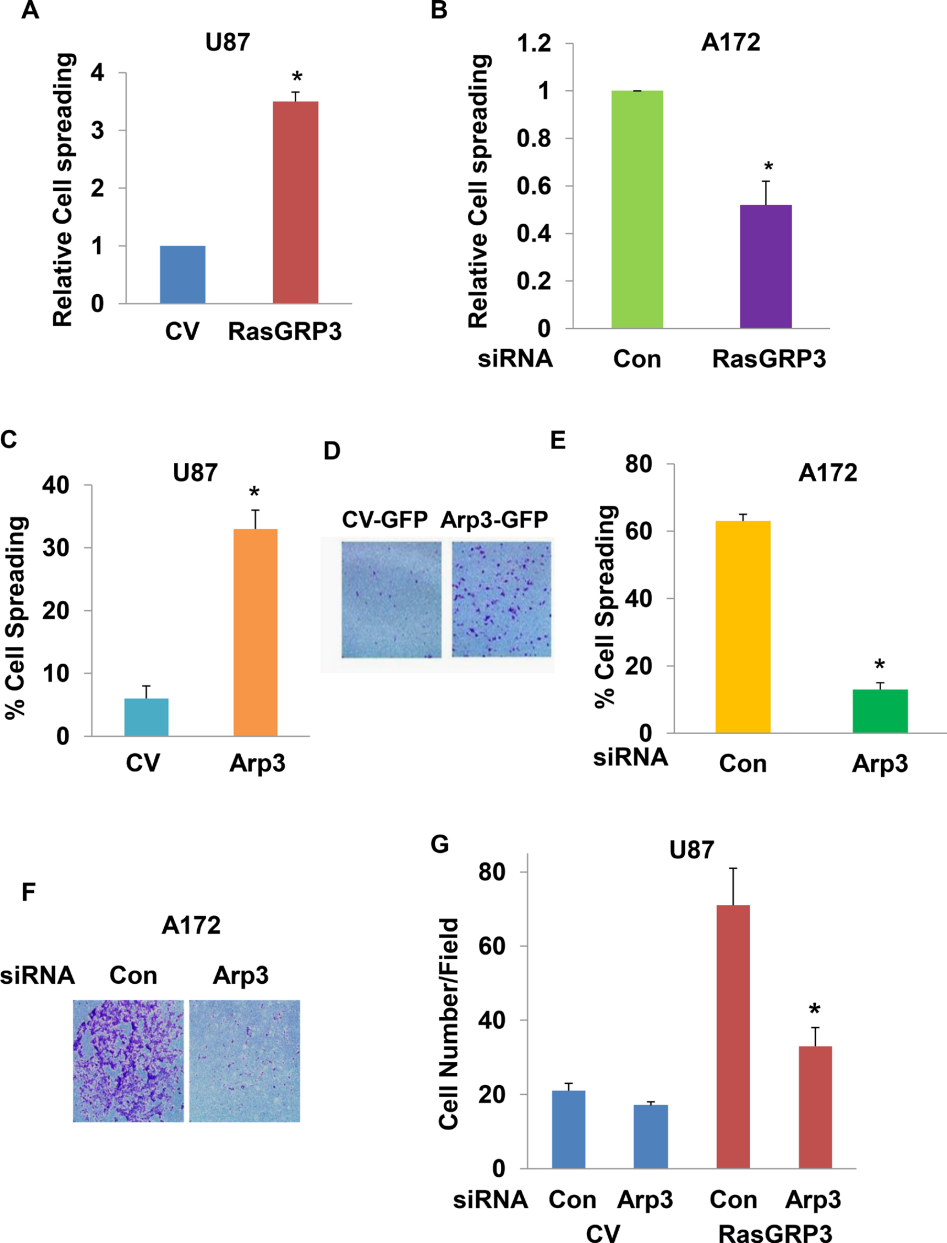
Arp3 plays a role in the effects of RasGRP3 on cell spreading and migration Spreading of U87 cells transfected with a CV or with RasGRP3 **(A)**, spreading of control and RasGRP3 siRNA transfected A172 cells **(B)**, spreading of U87 cells transfected with a CV or with Arp3 **(C)** or spreading of control and Arp3 siRNA transfected cells **(E)** were determined in trypsinized cells plated onto fibronectin-coated plates. After 30 min, multiple fields were imaged and cells were counted as described in Methods. Migration assays were determined for U87 cells transfected with a CV or Arp3 **(D)**, for A172 cells transfected with control or Arp3 siRNAs **(F)**, or for stable clones of RasGRP3 overexpressing U87 cells transfected with control or Arp3 siRNAs **(G)**. Cell migration was assayed using transwell migration assay. Each experiment represents triplicate determinations. The results represent the means ± SE of three independent experiments (A, B, C, E and G). *P < 0.001, compared with the appropriate controls or represent one of four experiments with similar results (D and F).

The Arp2/3 complex is known to play an important role in actin polymerization and cell spreading. To further study the functional interaction of RasGRP3 and Arp3, we first examined whether RasGRP3 affects cell spreading and if Arp3 mediates this effect. Cell spreading was assessed in RasGRP3-transfected cells by re-plating the cells on fibronectin following trypsinization. Overexpression of RasGRP3 in U87 cells increased cell spreading (Fig. 6A), whereas, silencing of RasGRP3 in A172 cells decreased this process (Fig. 6B).

To determine if Arp3 mediates the effects of RasGRP3 on glioma cell spreading and migration, we first examined Arp3 effects in glioma cells by overexpressing it in U87 cells and silencing it in the A172 cells. As presented in Fig. 6C and 6D, overexpression of Arp3 increased the spreading (Fig. 6C) and migration of U87 cells (Fig. 6D) and its silencing decreased the spreading (Fig. 6E) and migration of the A172 cells (Fig. 6F). Importantly, silencing of Arp3 abrogated the increased migration of the RasGRP3 overexpressing U87 cells (Fig. 6G). Silencing of Arp3 in the A172 cells or its overexpression in the U87 cells did not affect Ras or Rap activities in the A172 cells or that of the downstream signaling pathways, AKT and Erk1/2 (data not shown). Thus, these results demonstrate a functional significance of the interaction between RasGRP3 and Arp3 and indicate that the association with Arp3 plays a role in the effects of RasGRP3 on glioma cell migration, in a Ras-independent manner.

## DISCUSSION

Despite recent advances in therapeutic approaches for the treatment of GBM, the survival of patients diagnosed with these tumors remains dismal. This is mainly attributed to the high level of infiltration and treatment resistance of residual GSCs, which eventually results in tumor recurrence. Recently, GBM have been classified into several subtypes based on gene expression profile with the two subtypes, proneural and mesenchymal, being the most robust and generally consistent among the classification schemes [28]. The mesenchymal GBM are considered more infiltrative and radiation resistant [28, 29].

Activation of Ras signaling has been reported as an important pathway for the progression of human glioma; however, activating Ras mutations are rare in these tumors (2%, according to TCGA data) [23]. Therefore the increased activation of the Ras signaling pathway in GBM may be attributed to upstream pathways such as constitutively activated tyrosine kinase receptors, decreased RasGAP expression/activation or increased activation of GEFs, which may be deregulated in these tumors [22]. Here, we examined the expression and function of RasGRP3, which has been reported to act as a GEF for Ras and Rap1. We found that RasGRP3 was expressed in GBM specimens, in glioma cells and GSCs and its expression was higher in the tumor compared to the normal cells. Moreover, the expression of RasGRP3 was higher in the mesenchymal GBM subtype and high levels of RasGRP3 in GBM specimens were inversely correlated with patient survival.

RasGRP3 belongs to the DAG/PMA superfamily and has been reported to act as a GEF of both Ras and Rap1in various tissues and tumors. We found that RasGRP3 contributed to activation of the Ras pathway in glioma cells using both overexpression and silencing experiments. Overexpression of RasGRP3 in glioma cells that express low levels of this protein increased both Ras and Rap1 activity whereas silencing of endogenous RasGRP3 expression in glioma cells that express high levels of this protein decreased Ras but not Rap1 activation, suggesting that RasGRP3 might have differential affinity for these small GTPases in various glioma cells. In addition to its effects on the activation of Ras proteins, RasGRP3 also increased the activity of AKT and ERK1/2, two of the effector pathways downstream of Ras. Finally, RasGRP3 also partially mediated the induction of Ras activation by PMA indicating that RasGRP3 plays an important role in the integration of DAG-dependent signaling and the Ras pathway.

Additional GEFs have been reported to be overexpressed in GBM and to play a role in glioma cell migration and invasion [30]. Thus, Dock180, a GEF that activates RAC1 has been shown to be overexpressed in GBM specimens and to contribute to the invasion of these cells [31]. The Rho family GEFs, VAV3 and Trio, regulate the invasive phenotype of glioma cells [32], and a recent study identified the two GEFs, Ect2 and Trio, as activators of various Rho GTPases including cdc42 and Rac1 downstream of Tweak and as regulators of cell migration [33].

One of the factors that contribute to treatment failure in GBM is the presence of residual infiltrative cells that prevent complete tumor resection and exhibit resistance to current therapies. Thus, identification of pathways that promote glioma cell migration is of utmost importance. Using glioma cell lines expressing different levels of RasGRP3, we demonstrated that RasGRP3 regulated cell migration and invasion. Thus, silencing RasGRP3 expression decreased glioma cell migration and invasion, whereas overexpression of RasGRP3 increased these processes. The effect of RasGRP3 on glioma cell migration is in accordance with its increased expression in the mesenchymal GBM subtype that is characterized by increased infiltration. The mesenchymal transformation of GBM has been associated with the activation of transcription factors such as SNAIL, ZEB1, STAT3 and CEBP/β. Recent studies have also demonstrated that the Ras/MAPK pathway plays a role in promoting the mesenchymal transformation of GBM upstream of SNAIL. Studies are currently being performed to further delineate the role of RasGRP3 in the mesenchymal transformation of GBM and whether its effects on cell migration are associated with this process.

GBM exhibit an increased Ras activity [23, 34] and Ras controls key cellular processes such as differentiation, cytoskeleton arrangement, cell migration, and cell survival and death [35–38]. Three major Ras-mediated signaling pathways include the Raf/MAP kinase pathway that controls cell proliferation, the phosphatidylinositol 3-kinase (PI3K)/Akt pathway that mediates cell survival and cytoskeleton arrangement [39, 40] and the Ral-GDS/Ral pathway that participates in cell proliferation, motility, and cytoskeletal arrangement [41, 42]. In addition, Ras can regulate the activity of small G proteins that belong to the family of Rho GTPases, which also participate in cytoskeleton arrangement, cell cycle control, and gene expression [43]. It was reported that a Ras inhibitor (FTS) can avert the transformation of glioma cells by inhibiting both their migration and anchorage-independent proliferation. FTS inhibits the motility of glioma cells by down-regulating the PI3K pathway and shifting the balance between the activities of Rho- and Rac-GTPases [44]. We showed that a dominant negative form of Ras markedly reduced the migration of RasGRP3 overexpressing cells, suggesting a crucial role for Ras in RasGRP3-mediated cell migration. In contrast, although RasGRP3 increased the phosphorylation of AKT in glioma cells, the suppression of AKT activity in RasGRP3-overexpressing U87 cells did not abrogate the increased cell migration induced by RasGRP3, whereas it did inhibit the migration of the CV cells. These results indicate that the increased phosphorylation of AKT induced by RasGRP3 does not play a role in the effect of this protein on glioma cell migration. Further experiments determining the role of Rac and Rho in RasGRP3-induced cell migration, as well as analysis of other downstream effectors such as Erk1/2 are needed to further define the mechanisms of RasGRP3-induced glioma cell migration via Ras activation.

We identified Arp3, as a novel interacting protein of RasGRP3 in glioma cells. Arp3 is a major component of the Arp2/3 complex which regulates actin polymerization, lamellipodia formation and cell migration [45]. We found that Arp3 was highly expressed in GBM specimens and GSCs. High expression of Arp3 predicted poor patient survival, and similar to the expression of RasGRP3, Arp3 was also expressed in higher levels in the mesenchymal GBM subtype. To the best of our knowledge this is the first report of the expression and function of Arp3 in GBM and its association with RasGRP3 in the regulation of glioma cell migration. Reports of the expression and role of Arp3 in other tumors demonstrate that Arp2/3 acts as a prognostic factor and its expression is correlated with the increased motility and invasion of colorectal, adenocarcinomas and gastric tumors [46, 47].

We found that Arp3 regulated the spreading and migration of glioma cells. Moreover silencing of Arp3 abrogated the increased cell spreading and migration of cells overexpressing RasGRP3. Thus, Arp3 is functionally associated with RasGRP3 and plays a role in the effects of this protein on cell spreading and migration.

In summary, we demonstrate that RasGRP3 plays a major role in the activation of the Ras pathway in glioma cells. We demonstrated an important role of RasGRP3 in the regulation of glioma cell migration and invasion and identified Ras activation, but not that of AKT, as a partial mediator of this effect. In addition we identified Arp3 as a novel interacting protein of RasGRP3 and as a mediator of its effects on cell spreading and migration.

Recent studies reported that a high RasGRP3 expression contributed to the oncogenic characteristics of prostate and melanoma cancer cell lines [20, 21]. These observations, as well as our results, emphasize the importance of RasGRP3 as a positive regulator of Ras and the oncogenic potential of different tumors via activation of the classical Ras signaling pathways in addition to novel pathways such as Arp3. Thus, RasGRP3 acts in Ras-dependent and Arp3-dependent mechanisms and serves as an important link between DAG, the Ras signaling pathways and actin polymerization and may represent an important therapeutic target in GBM.

## MATERIALS AND METHODS

### Materials

An affinity-purified monoclonal anti-FLAG antibody, leupeptin, aprotinin, phenylmethylsulfonyl fluoride (PMSF), and sodium vanadate were obtained from Sigma Chemical Co. (St. Louis, MO) and anti-pAKT, AKT, phosphorylated ERK1/2, ERK1/2, and RasGRP3 antibodies were obtained from Cell Signaling Technology (Beverly, MA). Anti-Ras and anti-Rap1 antibodies were purchased from BD Biosciences (San Jose, CA). The AKT kinase-dead mutant vector was obtained from Addgene. The H-Ras dominant negative mutant vector was a gift from Professor Raymond Mattingly from Wayne State University (Detroit, MI). The pFLAG-RasGRP3 with an N-terminal FLAG tag was described previously [48].

### Cell culture, transfections and clone selection

U87 and A172 glioma cells were originally obtained from the American Type Culture Collection (Manassas, VA). Cells were cultured at 37ºC with CO_2_ and maintained in DMEM containing 10% (v/v) fetal bovine serum (FBS), 4 mM glutamine, 100 IU/ml penicillin, and 100 μg/ml streptomycin. Primary cultured glioma tumor cells were obtained from Hermelin Brain Tumor Center, Henry Ford Hospital (Detroit, MI, USA) with written consent in accordance with the local institutional ethics committee. To generate RasGRP3 over-expressing stable clones, we used the human glioma U87 cells which do not express this protein. Cells were transfected with either a control vector or a FLAG-tagged expression vector encoding RasGRP3 by electroporation using the Nucleofector device, protocol number U29 (Amaxa Biosystems, Gaithersburg, MD). Stable clones were isolated using selection medium containing 750 μg/ml of geneticin (Life Technologies, Gaithersburg, MD). Isolated clones were then cultured and expanded. Cell lysates were subsequently assayed for expression of RasGRP3 protein to select for RasGRP3-producing clones.

### Real-time RT-PCR

Total RNA was extracted using an RNeasy midi kit according to the manufacturer's (Qiagen, Germantown, MD) instructions. The reverse transcription reaction was carried out using 2 μg total RNA as described for the RT-PCR analysis. The cDNA samples were then used for real-time PCR (Invitrogen, Grand Island, NY). The following primers were used: RasGRP3 forward, TCTTGGTTTGATTCGTATGA; RasGRP3 reverse, TTTTTTCCTCTGTGTGACTC; S12 forward, TGCTGGAGGTGTAATGGACG; s12 reverse, CAAGCACACAAAGATGGGCT. The PCR conditions were described previously [27].

### Western blot analysis

Cell lysates and Western blot analysis were performed as previously described [49]. Equal protein loading was examined using Ponceau S staining or probing the membrane with anti-actin antibody.

### Transwell migration assay

Cell migration was assayed using a transwell culture chamber containing polycarbonate membrane inserts with 8-μm pore size (Cell Biolabs, Inc., San Diego, CA) as previously described [25]. Experiments were carried out 72 hrs after siRNA transfection. Cells were briefly incubated with trypsin to obtain a single-cell suspension, and 3 × 10^4^ cells in serum-free DMEM were added to the upper chamber. The bottom chamber was filled with 600 μl of medium, and the assembly was incubated at 37ºC for 4 hrs to allow cell migration. The cells were removed from the top of the membrane and the migratory cells were stained and quantified. In each experiment, three chambers were used for each experimental group.

### Matrigel invasion assay

Invasion of cells through matrix membranes was determined using 24-well BD invasion chambers (8-μm pore size with polycarbonate membrane, BD Biosciences) according to the manufacturer's instructions and as previously described [27].

### Cell spreading assay

Cell spreading assays were performed as described [50]. Briefly, cells were trypsinized, pelleted, resuspended in serum-free media and incubated at 37°C for 1 hr with gentle agitation. The cells were allowed to spread on the fibronectin-coated (10 μg/ml) plates and were visualized after 30 min. Spreading cells were defined as cells with irregular morphology and lacking phase brightness, whereas non-spreading cells were rounded and appeared phase-bright under the microscope. Multiple fields were imaged and ~ 200 cells were monitored and counted for each experiment. Three independent experiments were performed for each condition.

### Spheroid confrontation assay

The spheroid confrontation assay was performed as previously described [27]. Briefly, fetal rat brains were obtained from Sprague-Dawley rats at embryonic day 18 under a protocol approved by the Henry Ford Hospital Animal Care and Use Committee. Single-cell suspensions were made by digestion of whole brains in 0.1% trypsin/HBSS. Cell suspensions were cultured on agar-coated plates for 3 weeks to allow for the development of brain aggregates. Glioma cells were also cultured on agar-coated plates for 2 weeks to allow for the development of spheroids. Before the experiment, U87 cells were labeled with the Green Cell Tracker and the brain aggregates were labeled with the Red Cell Tracker (Invitrogen) to allow for the visualization of confrontations.

One aggregate and one spheroid were then transferred to a single agar-coated well of a 48-well culture plate and confronted mechanically. Confrontations were visualized after 72 hrs using an Olympus fluorescent microscope. Green and red fluorescence was visualized separately and then merged to visualize invasion.

### Small interfering RNA transfection

Small interfering RNA (siRNA) duplexes for RasGRP3 were synthesized and purified by Dharmacon (Lafayette, CO). A scrambled sequence was used as a negative control. Transfection of siRNAs was done using siIMPORTER (Upstate Group, Lake Placid, NY) according to the manufacturer's instructions. Experiments were performed 72 hrs after transfection.

### Analysis of GTP-bound Ras and Rap1

To determine the effects of RasGRP3 on Ras activity, the level of Ras-GTP was compared to the level of total Ras. Ras-GTP was determined by the glutathione *S*-transferase-Ras binding domain of Raf (RBD) pull-down assay. Pull-down of GTP-bound Ras was performed by incubating the cell lysates with GST fusion protein corresponding to the Ras-binding domain bound to glutathione-agarose at 4ºC for 30 min. Beads were washed twice with pull-down lysis buffer; the protein bound to the beads was eluted with Laemmli buffer and analyzed for the amount of GTP-bound Ras by immunoblotting using a Ras monoclonal antibody (BD Biosciences). Active Rap1 pull-down and detection were assayed in similar fashion by using a Rap1 activation kit from Pierce Biotechnology (Rockford, IL).

### Immunofluorescence staining

Cells were fixed in 4% paraformaldehyde (PFA) for 15 min and, after blocking with staining buffer (2% BSA and 0.1% Triton X-100 in PBS), were incubated with the anti-RasGRP3 antibody followed by incubation with an anti-rabbit Alexa Fluor 488 antibody. Cells were mounted using FluoroGuard antifade reagent and viewed and photographed using confocal microscopy. GFP and CFP transfected cells were analyzed similarly.

### Co-immunoprecipitation

Cells transfected with a control vector (CV) or RasGRP3 were used in this study. The samples were pre-absorbed with 25 μl of protein A/G-Sepharose (50%) for 10 min and immunoprecipitation was performed using 4 μg/ml anti-FLAG at 4°C for 1 hr and then incubated with 30 μl of A/G-Sepharose for an additional hour or overnight. After three washes, the pellets were resuspended in 40 μl of SDS sample buffer and boiled for 5 min. The entire supernatant was subjected to Western blotting.

### Affinity pull-down assay

Cell lysates from CV or RasGRP3 overexpressing cells were immunoprecipitated with anti-FLAG antibody. Analysis of excised in-gel digested bands was carried out by using a LC-nano MS/MS spectrometer (NextGen Sciences, Ann Arbor, MI). The sequences of individual peptides were identified by using the Mascot algorithm to search and correlate the MS/MS spectra with amino acid sequences in the protein database. Various proteins were detected by pull-down as potential RasGRP3-interacting proteins. Arp3 was identified as a band between 50–37 kDa based on its predicted size (47 kDa) in the stained gel. Details of the additional proteins that were identified will be described elsewhere.

### REMBRANDT data analysis

All glioma patient data were publicly available in de-identified form and obtained from the NCI Repository for Molecular Brain Neoplasia Data (REMBRANDT) database (https://caintegrator.nci.nih.gov/rembrandt/), using the data available on April 22, 2013. The correlation between RasGRP3 or Arp3 expression and overall patient survival was analyzed on 343 glioma patient samples. Although all of the patient samples are represented in the Kaplan–Meier Survival Plot, not all of the patients in the database were identified for glioma subtype. Thus, only those patients that were positively identified as having astrocytoma or GBM were used in the analysis for calculating the percentage of patients with upregulated, intermediate or downregulated RasGRP3 or Arp3 expression. The differences between groups were analyzed by log-rank *P* value.

### TCGA data analysis

Global gene expression was measured using the Agilent G4502A platform (G4502A-07-1, G4502A-07-2) as described elsewhere [26]. Probe-level RMA-processed data (level 2) were downloaded from the TCGA Data Matrix for 595 tumor samples (June 27, 2013) and 10 non-tumor samples (August 17, 2013) [47]. Sample files were merged on probe ID and standardized in R using the limma package NormalizeBetweenArrays function (cyclic loess, method='affy’). For consistency, only samples for which the origin of tissue was the brain, no prior tumor was recorded, and the histopathology was noted to be untreated primary (de novo) GBM were retained for this study. For cases with multiple tumor samples assayed, only the first sample was used as sorted alphanumerically by patient bar code.

The genes of interest (RASGRP3|25780 and ACTR3|10096) were mapped to the Agilent ReporterID codes in the array design file (G4502A-07-2). Molecular classification was taken from TCGA [51].

### Statistical analysis of TCGA data

The results are presented as the mean values ± SD. The data of patient specimens are presented graphically with median and interquartile range noted. Data were analyzed using analysis of variance or a Student's *t*-test. Correlation was assessed with the Pearson's correlation coefficient and tested against a correlation of zero (no correlation). The squared Pearson coefficient, or coefficient of determination (R^2^) for a single predictor regression, is given on the scatter plots. Kaplan-Meier analysis was used to produce survival curves with differences tested between groups by the log-rank test. Data were analyzed on a log 2 scale as appropriate.
